# Prominent ^18^F-FDG Uptake in the Adrenal Gland after Contralateral Adrenalectomy in a Known Case of Adrenocortical Oncocytic Carcinoma

**DOI:** 10.22038/AOJNMB.2022.67950.1472

**Published:** 2023

**Authors:** Rakan Al-Rashdan, Mohammed Aljaberi, Ali Mohamedkhair, Akram Al-Ibraheem

**Affiliations:** Department of Nuclear Medicine, King Hussein Cancer Center, Amman, Jordan

**Keywords:** Adrenocortical carcinoma, ^18^F-FDG PET/CT, Mitotane, Adrenalectomy

## Abstract

Adrenocortical carcinoma (ACC) is a rare type of cancer that is associated with a high rate of recurrence and poor prognosis. The main diagnostic approaches to adrenocortical cancer include CT scan, MRI and the promising role of ^18^F-FDG PET/CT. The main therapeutic approaches include radical surgery of local disease and recurrences, as well as adjuvant mitotane therapy.

The evaluation of adrenocortical carcinoma (ACC) could be difficult by using ^18^F-FDG PET/CT in view of the significant association between the ^18^F-FDG uptake and ACC. At the same time, not all adrenal glands with ^18^F-FDG uptake are considered to be malignant, so awareness of these various findings is substantial for ACC management, especially with limited data regarding the role of ^18^F-FDG PET/CT in ACC post-operative settings.

This report discusses the case of a 47-year-old man with a history of left adrenocortical carcinoma who underwent adrenalectomy and received adjuvant mitotane therapy. 9 months after the surgery, a follow-up ^18^F-FDG PET/CT scan showed that the ^18^F-FDG uptake was prominent in the right adrenal gland without corresponding abnormal CT scan findings.

## Introduction

 Adrenocortical carcinoma (ACC) is a rare tumor with an incidence of 1 per million people. It is associated with a high recurrence rate and poor prognosis; radical surgery of the primary tumor as well as of the local and distant recurrences is the only curative approach. Mitotane therapy is an effective adjuvant treatment for patients with ACC because it has delayed-onset adrenal-specific cytotoxic effects. Metastasis to the lung, liver, and lymph nodes is common, while metastasis to bone is less frequent. Imaging modalities play a central role in primary tumor detection, staging, and extent of recurrence in ACC. Although the imaging diagnostic tools for ACC are mainly based on using computed tomography (CT) and magnetic resonance imaging (MRI). Additionally, There have been studies published in the past that argue the role of ^18^F-FDG PET/CT as an emerging and promising tool in the management of ACC (1-5).

***Case Report***

 A 47-year-old male patient presented with hypertension refractory to several drugs. Upon diagnostic workup, an abdominal CT showed an unexpected mass on the left adrenal gland. Further tests showed that the urine and blood both had a lot of cortisol, which confirmed the diagnosis of Cushing syndrome.

 The patient had surgery to remove the left adrenal gland. Histopathological correlation confirmed the presence of adrenocortical carcinoma. The pathology report showed that the tumor size was 17×12×12 cm^3^, the tumor weight was 1370 grams, wide areas of necrosis were present with no capsular or vascular invasion, and the mitotic figures were less than 5/50 HPF. Melan-A and Synaptophysin proteins were found in tumor cells.A small number of cells were positive for inhibin. It is noteworthy that all the results are negative for EMA, chromogranin, and PAN-CK. The Ki-67 index was also below 5%. Adjuvant mitotane therapy was initiated based on the findings of the oncocytic variant.

**Figure 1:**
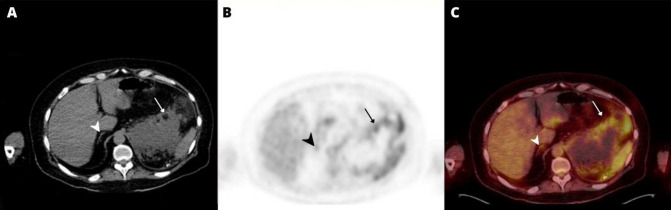
Axial CT (**A**), corresponding PET (**B**), and fused PET/CT (**C**) images revealed an irregular soft tissue mass of 14 cm in the left suprarenal region with mild non-specific FDG uptake (**arrows**), which was confirmed by biopsy to be negative for malignancy afterward, as well as normal morphology and metabolic activity of the right adrenal gland (**arrowheads**)

 A contrast-enhanced abdominal CT scan taken one month after a left adrenalectomy showed a left suprarenal mass that was heterogeneously enhanced and suspicious for metastatic deposits versus post-operative changes. In order to rule out residual or recurrent disease, an ^18^F-FDG PET/CT was performed ten days after the CE CT was originally conducted. After administration of 289 MBq of ^18^F-FDG, a PET/CT scan was performed. The ^18^F-FDG PET/CT scan showed an irregular soft tissue mass of 14 cm in the left suprarenal region with mild non-specific ^18^F-FDG uptake, which is considered a post-operative change as there is no significant FDG uptake also biopsy-proven to be negative for malignancy afterward. Figure 1 shows the right adrenal gland, which has normal structure and ^18^F-FDG activity.

**Figure 2:**
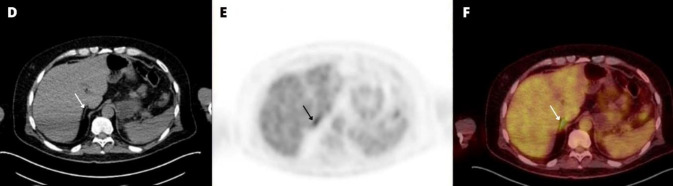
Axial CT (**D**) showed a normal-sized right adrenal gland (**arrow**), while the PET (**E**) and fused (**F**) showed prominent right adrenal gland FDG uptake (**arrows**, SUV_max_=5.6) that was almost two times higher than the physiologic liver FDG activity

## Discussion

 This case report presents the case of a 47-year-old man with a history of left adreno-cortical carcinoma who underwent adrenal-ectomy and received adjuvant mitotane therapy. 9 months after the surgery, a follow-up ^18^F-FDG PET/CT scan showed prominent ^18^F-FDG uptake in the right adrenal gland without corresponding abnormal CT scan findings.

 There are limited data regarding the role of ^18^F-FDG PET/CT in post-operative settings of adrenocortical carcinoma. However, several studies indicate that ^18^F-FDG PET/CT has a promising role in staging, therapy planning, local disease recurrence, and distant metastasis detection. Also, ^18^F-FDG PET/CT can play a complementary role with CT, especially in the presence of fibrosis or post-surgical changes (2). 

 Although there is no major recommendation to use ^18^F-FDG PET/CT after surgery, one study mentioned that some centers used ^18^F-FDG PET/CT for following up adrenocortical carcinoma removal surgeries at a 6-month interval period (7).

 It has been found that the sensitivity, specificity, and accuracy of both ^18^F-FDG PET/CT and contrast-enhanced CT at staging and recurrence were similar. One study found that the diagnostic abilities of ^18^F-FDG PET/CT were similar to or slightly higher than CT (6). 

 Another study showed that using PET in comparison to CT has a higher sensitivity for local disease recurrence (7).

 In 1957, the first reported usage of mitotane therapy in the treatment of adrenocortical cancers (8) was due to its adrenocytolytic effect (9). Mitotane therapy given after resection surgery was linked to a longer time without cancer coming back (10).

 Adrenocortical carcinomas appear as an enlarged mass with an inhomogeneous structure that displaces adjacent tissues on an unenhanced CT scan and as peripherally enhanced inhomogeneous masses with less central enhancement after intravenous contrast administration. At the same time, adreno-cortical cancers are considered ^18^F-FDG avid tumors on ^18^F-FDG PET/CT scans (11, 12). 

 Leboulleux et al. state that there is a significant association between the ^18^F-FDG uptake and the adrenocortical carcinoma mitotic rate (13). 

 However, not all adrenal glands with ^18^F-FDG uptake are considered to be malignant, as adrenal glands normally show ^18^F-FDG uptake but usually less than the liver (14). 

 There have been a few reported cases of increased ^18^F-FDG uptake greater than the liver within the normal adrenal gland, lasting up to 9 months after adrenalectomy for oncocytic variant ACC, with subsequent inhibition of cortisol production using mitotane therapy. This ^18^F-FDG uptake occurs most frequently during the first 6 months of mitotane treatment. Following the initial administration of mitotane therapy, patients are given high doses of corticosteroids, which is associated with high levels of Adrenocorticotropic hormone (ACTH). This increased ^18^F-FDG uptake is most likely due to the effect of ACTH stimulation. In such cases, this uptake is considered transient in 14-29% of cases and should not be concerning for malignancy (1, 14).

## Conclusion

 This case presents a transient high ^18^F-FDG uptake in the remaining adrenal gland 9 months after an adrenalectomy and subsequent mitotane therapy. This uptake is considered transient and occurs mostly in the first 6 months of mitotane treatment. It should not be worrisome for malignancy.

## Compliance with Ethical Standards

 Funding: The authors received no financial support for the research, authorship or publication of this article.

## Ethical approval

 All procedures performed in studies involving human participants were in accordance with the ethical standards of the institutional and/or national research committee and with the 1964 Helsinki declaration and its later amendments or comparable ethical standards.

## Informed Consent

 Informed consent was obtained from the patient for publication of his case/report and accompanying images.
